# Mediastinal lymph node metastasis model by orthotopic intrapulmonary implantation of Lewis lung carcinoma cells in mice

**DOI:** 10.1038/sj.bjc.6690178

**Published:** 1999-03

**Authors:** Y Doki, K Murakami, T Yamaura, S Sugiyama, T Misaki, I Saiki

**Affiliations:** Department of Pathogenic Biochemistry, Research Institute for Wakan-Yaku, Toyama Medical and Pharmaceutical University, 2630 Sugitani, Toyama, 930-0194, Japan; First Department of Surgery, Toyama Medical and Pharmaceutical University, 2630 Sugitani, Toyama, 930-0194, Japan

**Keywords:** lung cancer, animal model, mediastinal lymph node metastasis

## Abstract

This study is designed to establish a pulmonary tumour model to investigate the biology and therapy of lung cancer in mice. Current methods for forming a solitary intrapulmonary nodule and subsequent metastasis to mediastinal lymph nodes are not well defined. Lewis lung carcinoma (LLC) cell suspensions were orthotopically introduced into the lung parenchyma of C57/BL6 mice via a limited skin incision without thoracotomy followed by direct puncture through the intercostal space. The implantation process was performed within approximately 50 s per mouse, and the operative mortality was less than 5%. Single pulmonary nodules developed at the implanted site in 93% of animals and subsequent mediastinal lymph node metastasis was observed in all mice that formed a lung nodule after intrapulmonary implantation. The size of tumour nodule and the weight of mediastinal lymph node increased in a time-dependent manner. The mean survival time of mice implanted successfully with LLC cells was 21 ± 2 days (range 19–24 days). Histopathological analysis revealed that no metastatic tumour was detectable in the mediastinal lymph nodes on day 11, but metastatic foci at mediastinal lymph nodes were clearly observed on days 17 and 21 after implantation. Other metastases in distant organs or lymph nodes were not observed at 21 days after the implantation. Comparative studies with intrapleural and intravenous injections of LLC cells suggest that the mediastinal lymph node metastasis by intrapulmonary impantation is due to the release of tumour cells from the primary nodule, and not due to extrapulmonary leakage of cells. An intravenous administration of *cis*-diamine dichrolo platinum on day 1 after tumour implantation tended to suppress the primary tumour nodule and significantly inhibited lymph node metastasis. Thus, a solitary pulmonary tumour nodule model with lymph node metastasis approximates clinical lung cancer and may provide a useful basis for lung cancer research. © 1999 Cancer Research Campaign


*British Journal of Cancer* (1999) **79**(7/8), 1121–1126
© 1999 Cancer Research Campaign
Article no. bjoc.1998.0178

### Mediastinal lymph node metastasis model by orthotopic intrapulmonary implantation of Lewis lung carcinoma cells in mice

**Y Doki[1,2], K Murakami[1], T Yamaura[1], S Sugiyama[2], T Misaki[2] and I Saiki[1]**

[1]Department of Pathogenic Biochemistry, Research Institute for Wakan-Yaku, [2]First Department of Surgery, Toyama Medical and Pharmaceutical University, 2630 Sugitani, Toyama 930-0194, Japan

**Summary** This study is designed to establish a pulmonary tumour model to investigate the biology and therapy of lung cancer in mice. Current methods for forming a solitary intrapulmonary nodule and subsequent metastasis to mediastinal lymph nodes are not well defined. Lewis lung carcinoma (LLC) cell suspensions were orthotopically introduced into the lung parenchyma of C57/BL6 mice via a limited skin incision without thoracotomy followed by direct puncture through the intercostal space. The implantation process was performed within approximately 50 s per mouse, and the operative mortality was less than 5%. Single pulmonary nodules developed at the implanted site in 93% of animals and subsequent mediastinal lymph node metastasis was observed in all mice that formed a lung nodule after intrapulmonary implantation. The size of tumour nodule and the weight of mediastinal lymph node increased in a time-dependent manner. The mean survival time of mice implanted successfully with LLC cells was 21 ± 2 days (range 19–24 days). Histopathological analysis revealed that no metastatic tumour was detectable in the mediastinal lymph nodes on day 11, but metastatic foci at mediastinal lymph nodes were clearly observed on days 17 and 21 after implantation. Other metastases in distant organs or lymph nodes were not observed at 21 days after the implantation. Comparative studies with intrapleural and intravenous injections of LLC cells suggest that the mediastinal lymph node metastasis by intrapulmonary impantation is due to the release of tumour cells from the primary nodule, and not due to extrapulmonary leakage of cells. An intravenous administration of *cis*-diamine dichrolo platinum on day 1 after tumour implantation tended to suppress the primary tumour nodule and significantly inhibited lymph node metastasis. Thus, a solitary pulmonary tumour nodule model with lymph node metastasis approximates clinical lung cancer and may provide a useful basis for lung cancer research.

**Keywords:** lung cancer; animal model; mediastinal lymph node metastasis

Lung neoplasm, in place of gastric cancer, is the major cause of cancer mortality in Japan as well as in the USA (Travis et al, 1995). Despite the advances in diagnostic techniques for the early detection of lung cancer and the significant improvement in surgical procedures, the survival rate of lung cancer patients is poor even in the early stages of cancer as compared to the other malignant neoplasms. From a clinical point of view, one of the most troublesome impediments for treatment of lung cancer is the metastasis to mediastinal lymph nodes ($n = 2$ and $n = 3$ lung cancer) from the primary lesion (Jefferson et al, 1996). If metastases were inhibited by the present and/or new therapies, the prognosis of patients with lung cancer would improve. According to new TNM revisions (Mountain, 1997), it is difficult to say if the 5-year survival rate of $n \geq 2$ in lung cancer patients is an improvement, although a standard operative technique has been established to resect mediastinal lymph nodes (Vansteenkiste et al, 1997), except for aortic lymph node metastasis (Nakanishi et al, 1997). Therefore, biological approaches and studies, such as interference with lymph node metastasis or management of downstaging to the patients with lymph node metastasis, combined with surgery are required for lung cancer therapy. To do this, suitable animal models that conform to the clinical features are also necessary to search for novel therapies of lung cancer and to evaluate the efficacy of new drugs. However, there are few models for the formation of a solitary pulmonary nodule by orthotopic implantation of lung cancer cells and subsequent lymph node metastasis. In the present study, we attempted to establish a simple model for a solitary lung tumour and its lymph node metastasis by intrapulmonary implantation of Lewis lung carcinoma (LLC) cells in mice.

*Received 21 January 1998*
*Revised 26 May 1998*
*Accepted 25 June 1998*

*Correspondence to:* I Saiki, Department of Pathogenic Biochemistry, Research Institute for Wakan-Yaku, Toyama Medical and Pharmaceutical University, 2630 Sugitani, Toyama 930-0194, Japan

### MATERIAL AND METHODS

### Animals

Specific pathogen-free female C57BL/6 mice at 6 weeks old, were purchased from Japan SLC, Inc, Hamamatsu, Japan. They were maintained in the Laboratory for Animal Experiments, Research Institute for Wakan-Yaku Toyama Medical and Pharmaceutical University, under laminar air-flow conditions. All animals had free access to standard laboratory mouse food and water ad libitum. Housing was temperature controlled with a 12-h light and dark cycle. This study was conducted in accordance with the standards established by the Guidelines for the Care and Use of Laboratory Animals of Toyama Medical and Pharmaceutical University.

### Cells

LLC cells, originated spontaneously from mouse lung, were kindly provided by Dr K Takeda, Tohoku University, Tohoku, Japan. They were maintained as monolayer cultures in Eagle's minimal essential medium (EMEM) supplemented with 7.5% fetal bovine serum (FBS), vitamine solution, sodium pyruvate, non-essential amino acid, and L-glutamine (MA Bioproducts, Walkersville, MD, USA).

### Chemicals

CDDP (*cis*-diamine dichrolo platinum; randa®, 0.5 mg/ml) was purchased from Nippon Kayaku Co., Ltd, Tokyo, Japan. Matrigel® basement membrane matrix, possessing the ability to gel rapidly at 22–35°C, was purchased from Collaborative Biomedical Products, Bedford, MA, USA.

### Intrapulmonary implantation procedure

Log-phase cell cultures of LLC cells were harvested with 1 mM EDTA in phosphate buffered saline (PBS), washed three times with serum-free EMEM, and resuspended at a cell density of $5 \times 10^4$/ml in PBS containing 500 μg/ml of Matrigel®. Animals were anaesthetized with ether. The left chest was swabbed with 70% alcohol and a small skin incision to the left chest wall (approximately 5 mm in length) was made at about 5 mm tail side from the scapula. Subskin fat and muscles were separated from costal bones. On observing the left lung motion through the pleura, a 29 gauge needle attached to a 0.5 ml insulin syringe was directly inserted through the intercostal space into the lung to a depth of 3 mm. Tumour cells ($1 \times 10^3$) were suspended in 20 μl of PBS containing 10 μg of Matrigel® to prevent the suspension from leaking out of the lung, and were then injected into the lung parenchyma. A cotton-tipped applicator was pressed on the site of puncture as the needle was withdrawn to stop any bleeding. The skin incision was closed with a surgical skin clip. After confirming that the animals had recovered from bradycardia and possessed stable spontaneous respiration, they were returned to their cages.

### Macroscopic findings and histological study

Mice were sacrificed at various time periods (on days 11, 17 and 21) after tumour implantation, and the long and short diameters of the primary tumour mass and the weight of mediastinal lymph nodes for evaluating metastasis were measured manually. The tumour volume was calculated by the following formula: tumour volume ($mm^3$) = 1/2 × (long diameter) × (short diameter)$^2$. The lungs with a primary tumour nodule and mediastinal lymph nodes were excised for histological examination. In another experiment to evaluate the inhibitory effect of CDDP, mice were injected intravenously (i.v.) with CDDP at the clinical equivalent dose of 7 mg/kg (Nomura et al, 1996) after intrapulmonary implantation of LLC cells. T/C(%) of tumour growth was determined according to the formula: T/C(%) of tumour growth = (mean tumour growth of treated groups)/(mean tumour growth of untreated control groups) × 100.

### Statistical analysis

The statistical significance of differences between the groups was determined by applying the Student's two-tailed *t*-test or the Mann–Whitney *U*-test.

### RESULTS

### Mediastinal lymph node metastasis by orthotopic implantation of LLC cells

LLC cells ($1 \times 10^3$) were orthotopically injected through the intercostal space into the lung immediately after a small skin incision. The skin incision followed by intrapulmonary implantation of LLC cells was performed within approximately 50 s per mouse, and the operative mortality was less than 5%. Mean survival time of mice implanted successfully with LLC cells was 21 ± 2 days (range 19–24 days). In several experiments, tumours developed at the site of direct implantation in 93% (74/80) of animals, and all cases that formed a nodule in the lung had subsequent mediastinal lymph node metastasis. As shown in Figure 1, the implanted LLC cells developed a primary nodule in the lung which grew large as a function of time. The tumour volumes increased in a time-dependent manner, and were 0.75 ± 0.3, 34.7 ± 20 and 143 ± 134 $mm^3$ on days 11, 17 and 21 after the implantation, respectively (Figure 2). Tumours were visible to the naked eye in approximately 7 days. In addition, metastases to mediastinal lymph nodes were observed on days 17 and 21 after the implantation (Figure 1). A marked increase in the weight of the lymph nodes with a cobble stone-like appearance was macroscopically seen on day 21 (Figures 1 and 2). Similar to the tumour volume, the weight of resected mediastinal lymph nodes increased in a time-dependent manner (506 ± 213 and 1529 ± 759 mg on days 17 and 21, respectively). In these experiments, no unexpected tumour growth into the chest wall or pleural disseminations were seen.

### Histopathological observation

We next investigated the histopathological determination of the resected primary tumour and mediastinal lymph nodes on days 11, 17 and 21 after the implantation. Figure 3 shows that tumor cells proliferated uniformly and intimately all over the specimen on days 11 and 17, and central necrosis with bleeding in the nodule was observed on day 21. In addition, no metastatic tumour was seen in the mediastinal lymph nodes on days 11, but metastatic foci were clearly observed on days 17 and 21 after implantation (Figure 4). No metastatic nodule was macroscopically observed in distant organs and lymph nodes such as liver, spleen and cervical and supra/sub clavian lymph nodes, even on day 21 (moribund state) after implantation.

### Effect of CDDP on tumour growth and lymph node metastasis produced by intrapulmonary implantation of LLC cells

To evaluate the efficacy of anti-cancer drugs in this model, mice were injected i.v. with CDDP at the clinical equivalent dose of 7 mg/kg (Nomura et al, 1996) on day 1 or 10 after intrapulmonary implantation of LLC cells. The primary tumour volumes and the weight of mediastinal lymph nodes were measured on day 18 after implantation. Administration of CDDP on day 1 tended to suppress the primary tumour volumes as compared with the untreated control, whereas the weight of mediastinal lymph nodes was significantly inhibited by treatment with CDDP ($P < 0.01$) (Experiment 1, Figure 5). The tumour volumes and weight of lymph nodes in control and CDDP-treated groups were 128.7 ± 95.6 and 65.5 ± 82.3 $mm^3$, and 316 ± 226 and 97 ± 135 mg, respectively (Experiment 1, Figure 5). When CDDP was administered i.v. on

 © Cancer Research Campaign 1999

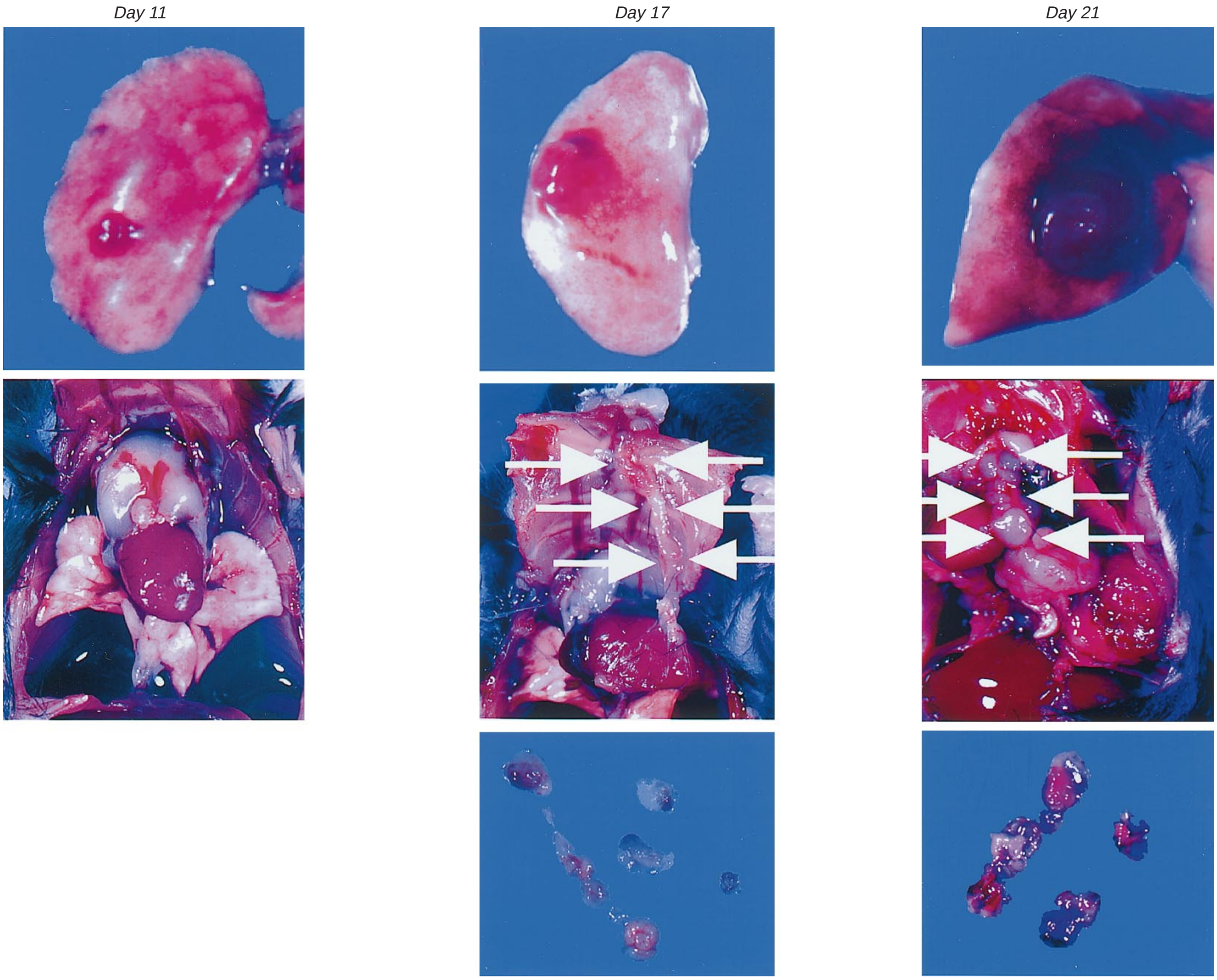

**Figure 1** Macroscopic findings of resected lung and mediastinum on various days after intrapulmonary implantation of LLC cells. The implanted tumour cells formed a pulmonary nodule in the lung and the tumour sizes increased with time. The metastases to mediastinal lymph nodes were visible on days 17 and 21 after implantation (→)

day 10 after tumour implantation, it did not affect the primary tumour volumes, but tended to inhibit the weight of lymph nodes although the effect was not significant (Experiment 2, Figure 5).

### DISCUSSION

In the present study, we have established a useful model for a solitary pulmonary tumor and subsequent metastasis to mediastinal lymph nodes by intrapulmonary implantation of LLC cells in syngeneic immunocompetent mice. An advantage of this model includes the simple and easy implantation procedure with a small skin incision at a predetermined site followed by direct puncturing through the intercostal space to lung parenchyma, without thoracotomy or intubation. The whole implantation process was performed within approximately 50 s per mouse, and the operative mortality was less than 5%. Tumours developed at the site of direct implantation in 93% of animals.

Several studies have shown that the biological behaviour of human tumour cells is influenced by the implantation site (Fidler, 1986), and that the orthotopic implantation of human tumour cells into relevant organs of nude mice can provide an in vivo model to study the biology and therapy of these cells (Fidler, 1986; McLemore et al, 1988). Orthotopic nude mouse or nude rat models have been developed for a number of human cancers including those of lung, colon, kidney and pancreas (Tan and Chu, 1985; Naito et al, 1986; McLemore et al, 1987; Morikawa et al, 1988). In particular, orthotopic models for human lung cancer have been reported by several investigators (McLemore et al, 1988, 1997; Wang and Hoffman, 1992); however, they have some problems such as the complicated procedures including thoracotomy (Wang and Hoffman, 1992) or intubation, long observation times and the necessity of immunosuppression by some agents (Yano et al, 1996) or irradiation (Howard et al, 1991) to increase the take-rate of tumours. Previous studies have shown that ectopic implantations such as i.v., subcutaneous (s.c.) or intrafootpad injections of lung cancer cells achieved the formation of secondary tumours in lung (Talmadge and Fidler, 1982; Brodt, 1986), although their use as a lung cancer model may not reflect the clinical course of primary lung cancer. Recently, Wang et al (1997) have shown a simple model in which chemically-induced sarcoma cells were

© Cancer Research Campaign 1999 

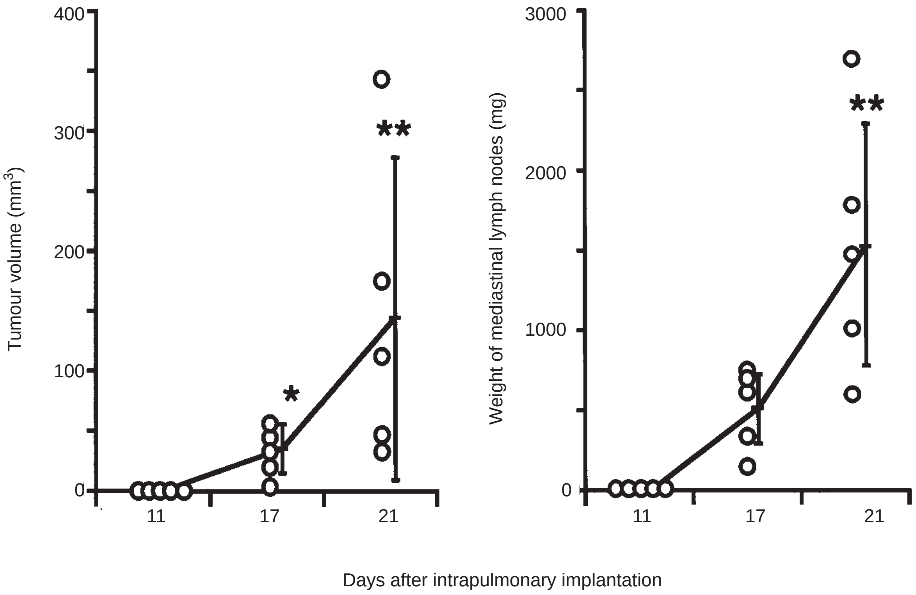

**Figure 2** Changes in tumour volumes and weights of mediastinal lymph nodes on various days after intrapulmonary implantation of LLC cells. Five mice per group were orthotopically inoculated with LLC cells ($1 \times 10^3$) and at various days later, the tumour sizes and weight of mediastinal lymph nodes were measured. **P* < 0.05; ***P* < 0.01

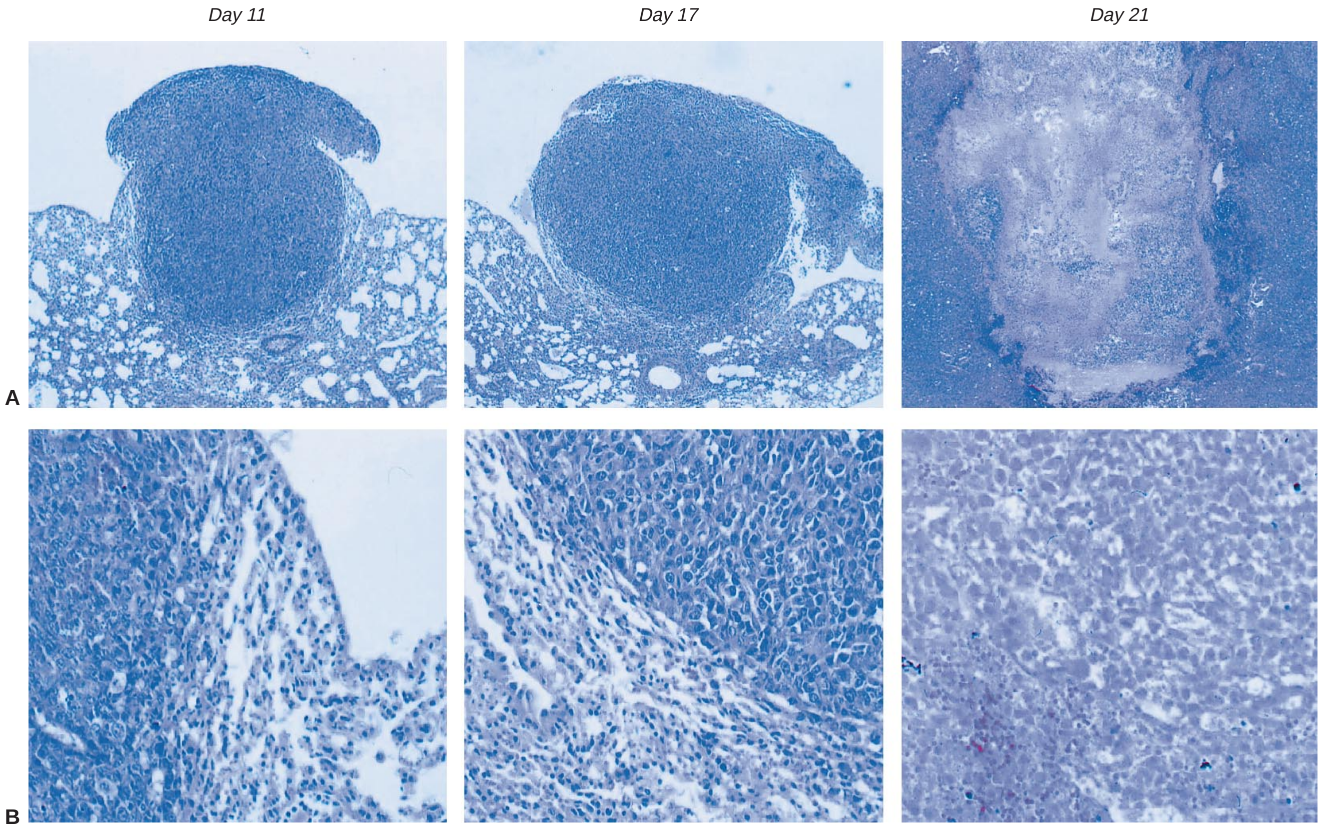

**Figure 3** Pathological findings of the pulmonary tumour nodule on various days after intrapulmonary implantation of LLC cells. Central necrosis was extensively observed in comparison with the proliferative growth in peripheral area of the nodule on day 21. (**A**) × 50, (**B**) × 250

directly implanted to the lung of thoracotomized Fisher-344 rat, but this is not an orthotopic implantation model.

Matrigel® has been used as a standard procedure for efficient formation of tumours, especially high take-rate by s.c. implantation of human tumours in athymic mice (Friedman et al, 1990, 1991). As shown in Figures 1 and 2, orthotopic implantation of LLC cells suspended in PBS containing Matrigel® resulted efficiently in the formation of a solitary tumour nodule, and the tumour volume

© Cancer Research Campaign 1999

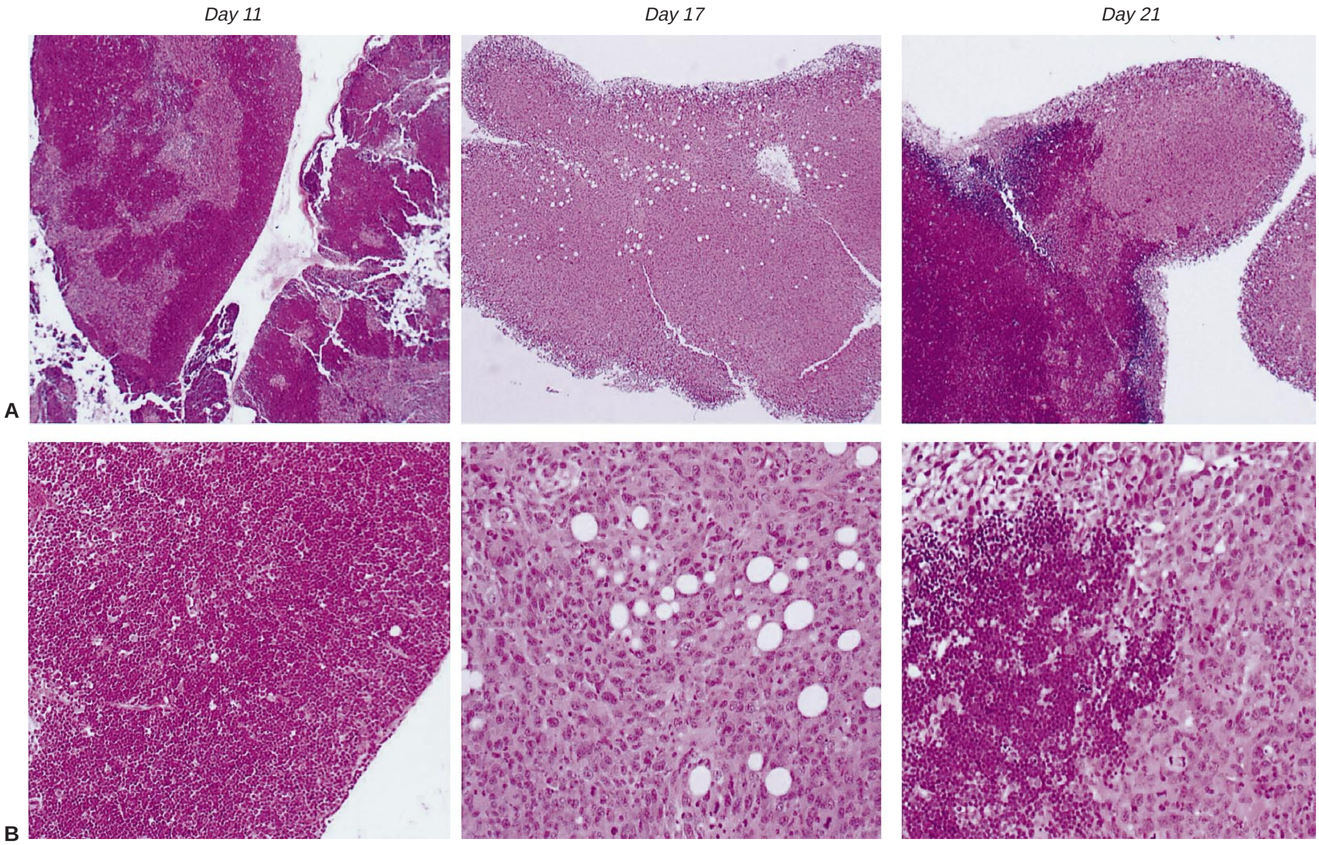

**Figure 4** Pathological findings of the mediastinal lymph nodes on various days after intrapulmonary implantation of LLC cells. No metastatic lesion was seen on day 11, whereas certain metastases were observed on days 17 and 21, central necrosis of tumour was also seen on day 21. (**A**) × 50, (**B**) × 250

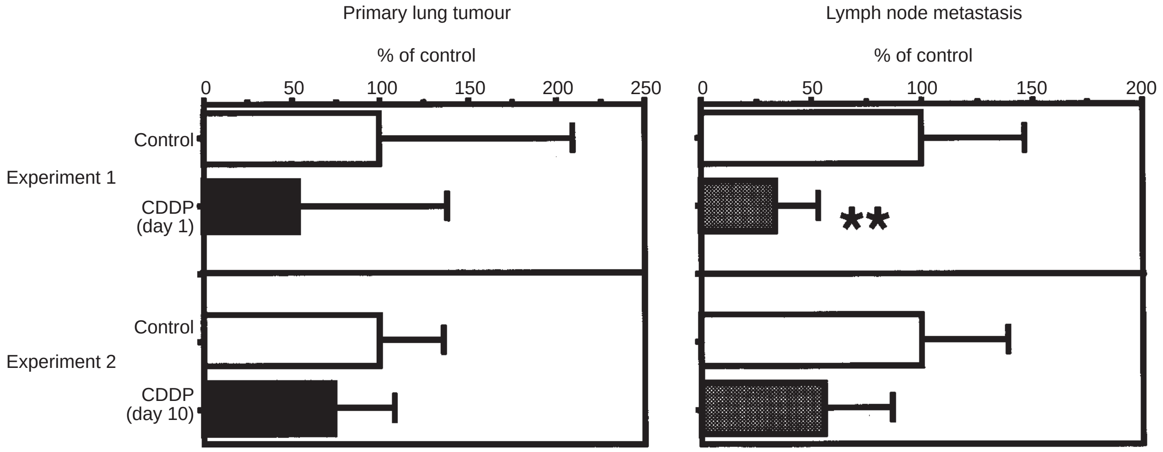

**Figure 5** Effect of CDDP on the growth of the orthotopically implanted tumour and metastasis to mediastinal lymph nodes. Seven or four C57BL/6 mice per group (Experiments 1 or 2) were injected with LLC cells ($1 \times 10^3$) suspended in PBS containing 10 µg Matrigel® into the left lung. CDDP (7 mg/kg) was given i.v. on day 1 (Experiment 1) or 10 (Experiment 2) after tumour implantation. Mice were sacrificed on day 18, and then primary tumour size and the weight of mediastinal lymph nodes were measured. $^{**}P < 0.01$ by Student's two-tailed *t*-test increase was time-dependent. Similarly, the increase in the weight of mediastinal lymph nodes was observed in a time-dependent manner. In our preliminary studies, intrapulmonary implantation of cell suspension containing dye as an indicator without Matrigel® caused rapid dispersion and extrapulmonary leakage of the dye within approximately 20 s after the injection, while the implantation of cell suspension with Matrigel® (500 µg/ml), which has the ability to gel rapidly at 22–35°C, showed clear localization of the dye at the injection site of lung. In addition, intrapulmonary or intrapleural injections of LLC cells without Matrigel® showed no formation of the solitary nodule on day 17, while pleural dissemination was observed (data not shown). Intravenous injection of

© Cancer Research Campaign 1999

tumour cells also caused the formation of multiple metastatic colonies in lung. On the other hand, metastases to mediastinal lymph nodes were observed by any injection route of the tumour cells. These results indicate that the suspending of tumour cells in Matrigel® resulted in the efficient formation of a solitary pulmonary nodule by implantation, and prevented the tumour cell suspension from leaking out of the lung, which would consequently lead to extrapulmonary tumour growth. Since pleural dissemination was not observed after intrapulmonary implantation of tumour cells with Matrigel®, which is different from the case of intrapleural injection, the metastasis to mediastinal lymph nodes would be due to the release of tumour cells from the primary nodule, and not due to extrapulmonary leakage of tumour cells.

Matrigel® has been shown to influence the behaviour and aggressiveness of tumours, such as promotion of tumorigenicity and invasiveness, drug resistance and induction of vascularization (Friedman et al, 1990, 1991; Yamamura et al, 1993; Bonfil et al, 1994; Ito et al, 1996). Mice bearing primary nodules in lungs showed 100% incidence of mediastinal lymph node metastasis. Thus, the co-injection with Matrigel® may lead to the enhancement of lymph node metastasis as well as the formation of primary tumour through the above mentioned properties of Matrigel®.

To investigate the usefulness of this model for evaluating anticancer drugs, we also examined the effect of CDDP as a major drug used for clinical chemotherapy on primary tumour growth and lymph node metastasis in our intrapulmonary implantation model (Figure 5). A single administration of the clinical equivalent dose of CDDP (Nomura et al, 1996) on day 1 after intrapulmonary implantation of LLC cells tended to suppress primary tumour growth and significantly inhibited metastasis to mediastinal lymph nodes. CDDP administration on day 10 did not affect primary tumour growth, but tended to inhibit lymph node metastasis. This indicates that the inhibition of mediastinal lymph node metastasis by CDDP may be due to the growth inhibition of both primary tumour and metastatic tumour in lymph nodes. However, further study will be needed to determine the timing of the administration and schedules for evaluating the exact efficacy. Our model using murine lung carcinoma cells in syngeneic immunocompetent mice may also be useful for evaluating the efficacy of some biological response modifiers which possess the immunomodulating properties.

Thus, our solitary pulmonary tumour nodule model with lymph node metastasis approximates clinical lung cancer. This procedure is simple and reproducible with a low implantation mortality and a high rate of solitary nodule development, because it involves only a small skin incision followed by direct puncturing through the intercostal space to the lung parenchyma, without thoracotomy or intubation. Therefore, this model may provide a useful basis for lung cancer research.

### ACKNOWLEDGEMENTS

This work was supported in part by Grants-in-Aid for Cancer Research from the Japanese Ministry of Education, Science, Sports and Culture (Nos 09254101 and 07273106). We thank Dr T Sakai, Second Department of Histopathology, Toyama Medical and Pharmaceutical University, for his pathological determination of specimens and Ms Kazuko Hayashi for her technical assistance.

### REFERENCES

Bonfi RD, Vinyals A, Bustuoabad OD, Llorens A, Benavides FJ, Gonzales-Garrigues M and Fabra A (1994) Stimulation of angiogenesis as an explanation of Matrigel®-enhanced tumorigenicity. *Int J Cancer* **58**: 233–239

Brodt P (1986) Characterization of two highly metastatic variants of Lewis lung carcinoma with different organ specificities. *Cancer Res* **46**: 2442–2448

Fidler IJ (1986) Rationale and methods for the use of nude mice to study the biology and therapy of human cancer metastasis. *Cancer Metastasis Rev* **5**: 29–49

Friedman R, Giaccone G, Kanemoto T, Martin GR, Gazdar AF and Mulshine JL (1990) Reconstituted basement membrane (Matrigel®) and laminin can enhance the tumorigenicity and the drug resistance of small cell lung cancer cell lines. *Proc Natl Acad Sci USA* **87**: 6698–6720

Friedman R, Kibbey MC, Royce LS, Zain M, Sweeney TM, Jicha DL, Yannelli JR, Martin GR and Kleinman HK (1991) Enhanced tumor growth of both primary and established human and murine tumor cells in athymic mice after coinjection with Matrigel®. *J Natl Cancer Inst* **83**: 769–774

Howard RB, Chu H, Zeligman BE, Marcell T, Bunn PA, McLemore TL, Muilvin DW, Cowen ME and Johnston MR (1991) Irradiated nude rat model for orthotopic human lung cancers. *Cancer Res* **51**: 3274–3280

Ito Y, Iwamoto Y, Tanaka K, Okuyama K and Sugioka Y (1996) A quantitative assay using basement membrane extracts to study tumor angiogenesis in vivo. *Int J Cancer* **67**: 148–152

Jefferson MF, Pendleton N, Faragher EB, Dixon GR, Myskow MW and Horan MA (1996) 'Tumour volume' as a predictor of survival after resection of non-small-cell lung cancer (NSCLC). *Br J Cancer* **74**: 456–459

McLemore TL, Liu MC, Blacker PC, Gregg M, Alley MC, Abbott BJ, Shoemaker RH, Bohlman ME, Litterst CC, Hubbard WC, Brennan RH, McMahon JB, Fine DL, Egglestone JC, Mayo JG and Boyd MR (1987) Novel intrapulmonary model for orthotopic progression of human lung cancers in athymic nude mice. *Cancer Res* **47**: 5132–5140

McLemore TL, Egglestone LC, Shoemaker RH, Abbott BJ, Bohlman ME, Liu MC, Fine DL, Mayo JG and Boyd MR (1988) Comparison of intrapulmonary, percutaneous, intrathoracic, and subcutaneous models for the propagation of human pulmonary and non-pulmonary cancer cell lines in athymic nude mice. *Cancer Res* **48**: 2880–2886

Morikawa K, Walker SM, Jessup JM and Fidler IJ (1988) In vivo selection of highly metastatic cells from surgical specimens of different primary human colon carcinoma implanted into nude mice. *Cancer Res* **48**: 6863–6871

Mountain CF (1997) Revisions in he international system for staging lung cancer. *Chest* **111**: 1710–1717

Nakanishi R, Osaki T, Nakanishi K, Yoshino I, Yoshimatsu T, Watanabe H, Nakata H and Yasumoto K (1997) Treatment strategy for patients with surgically discovered N2 non-small cell lung cancer. *Ann Thorac Surg* **64**: 342–348

Naito S, Von Eshenbach AC, Giavazzi R and Fidler IJ (1986) Growth and metastasis of tumor cells isolated from a human renal cell carcinoma implanted into different organs of nude mice. *Cancer Res* **46**: 4109–4115

Nomura T, Sakurai Y and Inaba M (1996) *The Nude Mouse and Anticancer Drug Evaluation*, pp. 29–45, Chemotherapy Publishers: Tokyo

Talmadge JE and Fidler IJ (1982) Enhanced metastatic potential of tumor cells harvested from spontaneous metastases of heterogenous murine tumors. *J Natl Cancer Inst* **69**: 975–980

Tan MH and Chu TM (1985) Characterization of the tumorigenic and metastasis properties of a human pancreatic tumor cell line (ASPC-1) implanted orthotopically into nude mice. *Tumor Biol* **6**: 89–98

Travis WD, Travis LB and Devesa SS (1995) Lung cancer. *Cancer* **75**: 191–202

Vansteenkiste JF, De Leyn PR, Deneffe GJ, Stalpaert G, Nackaerts KL, Lerut TE, Demedts MG and Leuven Lung Cancer Group (1997) Survival and prognostic factors in resected N2 non-small cell lung cancer: a study of 140 cases. *Ann Thorac Surg* **63**: 1441–1450

Wang HY, Ross HM, Ng B and Burt ME (1997) Establishment of an experimental intrapulmonary tumor nodule model. *Ann Thorac Surg* **64**: 216–219

Wang X, Pu X and Hoffman RM (1992) A new patient-like metastatic model of human lung cancer constructed orthotopically with intact tissue via thoracotomy in immunodeficient mice. *Int J Cancer* **51**: 992–995

Yamamura K, Kibbey MC, Jun SH and Kleinman HK (1993) Effect of Matrigel® and laminin peptide YIGSR on tumor growth and metastasis. *Semin Cancer Biol* **4**: 259–265

Yano S, Nishioka Y, Izumi K, Tsuruo T, Tanaka T, Miyasaka M and Sone S (1996) Novel metastasis model of human lung cancer in SCID mice depleted of NK cells. *Int J Cancer* **67**: 211–217

 *© Cancer Research Campaign 1999*
